# Sex differences in number processing: Differential systems for subtraction and multiplication were confirmed in men, but not in women

**DOI:** 10.1038/srep39064

**Published:** 2016-12-14

**Authors:** Belinda Pletzer

**Affiliations:** 1 Department of Psychology & Centre for Cognitive Neuroscience, University of Salzburg, Salzburg Austria

## Abstract

Neuroimaging studies suggest segregated neuronal systems underlying number magnitude processing (e.g. subtraction) and arithmetic fact retrieval (e.g. multiplication). While number magnitude processing is thought to rely on the intraparietal sulcus (IPS) bilaterally, arithmetic fact retrieval is thought to rely on the left angular gyrus (AG). However, evidence from brain damaged patients and brain stimulation challenges this view and suggests considerable overlap between the systems underlying number magnitude processing and arithmetic fact retrieval. This study investigates, whether sex differences in number processing can account for these conflicting findings. A subtraction and a multiplication task were administered to 40 men and 34 women in their luteal phase during functional MRI. Replicating previous studies in men, we found the IPS to be more strongly activated during subtraction than multiplication, and the AG to be more strongly activated during multiplication than subtraction. However, no differences between the two tasks were observed in women.

The triple-code model of number processing^1^ suggests segregated neuronal systems for the processing of number magnitudes (e.g. subtraction or number comparison) and the retrieval of arithmetic facts, (e.g. single digit addition and multiplication).

There is a large number of neuro-imaging studies pinpointing the processing of number magnitudes to the intraparietal sulcus (IPS), bilaterally, and the retrieval of simple multiplication facts to the left angular (AG) and supramarginal gyrus (SMG)^1^. Results from studies using different numerical tasks for number magnitude processing and arithmetic fact retrieval (e.g. subtraction vs. multiplication^2^, were corroborated by results from a study manipulating these two aspects of numerical abilities within the same task^3^. In a number bisection task, multiplicative items (e.g. 12_14_16) are part of a multiplication series and can be solved via arithmetic fact retrieval, whereas non-multiplicative items (e.g. 13_15_17) are not part of a multiplication series and require number magnitude processing. Non-multiplicative compared to multiplicative items lead to increased BOLD-response in the IPS. Multiplicative compared to non-multiplicative items however, lead to increased BOLD-response in the left AG.

The IPS is also a prominent activation site during spatial processing e.g.^4^,^5^, while the AG is also involved in verbal and memory processing e.g.^6^. These shared activation sites as well as behavioural evidence led to the assumption, that number magnitudes have a spatial representation^7^, and arithmetic fact retrieval is verbally mediated^1^.

Further support for the notion that number magnitude processing and arithmetic fact retrieval rely on dissociated neural systems comes from studies on brain damaged patients. Lesions to the IPS lead to selective impairment of subtraction, but preserved multiplication, whereas lesions to the left AG resulted in selective impairments of multiplication, but preserved subtraction^8^,^9^.

However, a specific impairment of multiplication fact retrieval has also been reported in cases without lesions to the left AG/SMG, whereas preserved multiplication performance was observed in a patient with severe lesions to the left AG/SMG^10^,^11^. Furthermore, in brain stimulation studies, parts of the left AG and IPS were identified as being essential for both subtraction and multiplication^12^,^13^,^14^. These findings challenge the idea of segregated neuronal systems for arithmetic fact retrieval and number magnitude processing.

The present study investigates, whether sex differences in the dissociation between the two systems could explain these conflicting findings. The idea that men and women might differ in the dissociability of the neuronal systems underlying number magnitude processing and arithmetic fact retrieval stems from the following observations.

First, neuroimaging studies pinpointing number magnitude processing to the IPS and arithmetic fact retrieval to the AG were originally performed on small mixed-sex samples e.g.^2^,^15^,^16^, or exclusively male samples e.g.^17^,^18^. Likewise, in the number bisection study of Wood *et al*.^3^ only male participants were recruited^3^. While newer studies with larger mixed-sex samples find similar results^14^,^19^,^20^,^21^,^22^,^23^,^24^,^25^,^26^,^27^,^28^,^29^, a replication of this dissociation for exclusively female samples is still missing. If the dissociation is strong in males, but absent in females, results observed in mixed-sex samples might be driven by males only.

Second, if number magnitude processing is related to spatial processing, while arithmetic fact retrieval is related to verbal processing^30^, inter-individual differences in spatial and verbal abilities should affect the different numerical abilities. Since men score reliably better than women in spatial tasks^31^,^32^,^33^,^34^, they should have an advantage compared to women in tasks requiring number magnitude processing, like number comparison, subtraction or non-multiplicative number bisection items. Vice versa, since women score reliably better than men in verbal and memory tasks^34^,^35^, they should have an advantage compared to men in tasks allowing for arithmetic fact retrieval, like single digit addition or multiplication or multiplicative number bisection items. However, Royer *et al*.^36^ report that men outperform women in the retrieval of arithmetic facts^36^, a finding we were recently able to replicate using a multiplication task^37^. Furthermore, men outperformed women in multiplicative number bisection items^38^. Thus, men outperform women in both number magnitude processing and arithmetic fact retrieval.

Third, also in the number bisection task^38^, only men show consistently better performance during multiplicative than during non-multiplicative items, which was accompanied by increased activation of the left AG/SMG during multiplicative items. In women, the same activation site did not display activation, but deactivation in response to number bisection items. Behavioural and BOLD-response differences in the AG/SMG between multiplicative and non-multiplicative items were either reversed (follicular phase: better performance, less deactivation in non-multiplicative items) or dependent on the numerical distance of items (luteal phase). Thus, in the number bisection task, performance and brain activation differs between number magnitude processing and arithmetic fact retrieval only in men.

Fourth, we vice versa, recently observed a strong relationship between performance measures during subtraction and multiplication in women, which was absent in men^37^. This suggests a stronger inter-dependence between performance during number magnitude processing and arithmetic fact retrieval in women than in men.

Fifth, while there are several possible explanations for the variations in lesion studies of the AG/SMG, it is interesting to note that van Harskaamp *et al*.’s patient with lesions to the left AG/SMG, but preserved multiplication ability, was female^10^, while Dehaene and Cohen’s patient with similar lesions, but an acquired inability to retrieve multiplication facts, was male^8^.

Taken together, sex could be an important factor explaining why some studies find the systems for number magnitude processing and arithmetic fact retrieval to be dissociated, while other studies find them to be overlapping. The question arises whether men and women differ in the extent to which arithmetic facts are actually retrieved from memory or to which arithmetic fact retrieval is indeed verbally mediated and vice versa for number magnitude processing and spatial abilities.

Therefore in the present study we employed both a subtraction task to assess number magnitude processing and a multiplication task to assess arithmetic fact retrieval in a sample of 43 healthy young men on the one hand and a matched sample of 39 healthy young women on the other hand during functional MRI. The aim was to replicate the dissociation of neural systems underlying subtraction and multiplication in men and identify, whether the same dissociation could be identified in women, or whether in women a stronger overlap between the two systems could be observed. In order to validate our findings, we also modulated two factors in each task that have previously been demonstrated to affect task performance.

## Materials and Methods

### Participants

43 healthy young men and 38 healthy young women participated in the study. All participants were right-handed Caucasians with German as their native language and had comparable educational status (students, who had passed their A-levels). All participants were free of medication and had no history of psychological, neurological or endocrinological disorders. No participant displayed brain tissue abnormalities on the structural MRI, but four participants (3 men, 1 woman) had to be excluded due to excessive head movements.

Since it had previously been demonstrated that brain activation during number processing is affected by menstrual cycle phase and hormonal contraceptive use^38^,^39^,^40^, only women, who had not been using hormonal contraceptives for the past six months were allowed to participate, in order to capture naturally occurring sex differences. All women had a regular menstrual cycle with a mean duration of 28.58 days (SD = 2.93 days, range: 21–35 days). Since sex differences are usually more pronounced when the female sex hormones estradiol and progesterone are high e.g.^41^, scanning sessions were scheduled in the mid-luteal cycle phase (3–10 days after ovulation, mean cycle day: 21.42 ± 3.62). Ovulation was determined as lying 14 days before the expected onset of the next period based on participants self-reports of the first day of their last period and their cycle duration over the last three cycles. The cycle phase was confirmed by follow-up reports of the onset of the next period and the analysis of estradiol and progesterone levels. Three women, who did not get their period on the expected date and had low progesterone levels at the day of testing, were excluded from the analysis. Thus, in total 40 men (mean age: 25.25 ± 4.71, range: 19–40 years) and 34 women (mean age: 25.56 ± 4.29, range: 18–36 years) were analyzed. Age did not differ between men and women (t_(72)_ = 0.29, p = 0.77, d = 0.06). General intelligence was screened after the scanning session using Raven’s advanced progressive matrices (APM)^42^ and did also not differ between men (mean IQ = 113.79 ± 11.62) and women (mean IQ = 114.88 ± 7.62; t_(52)_ = 0.40, p = 0.69, d = 0.11). [Note however that 20 participants (11 men, 9 women) did not complete the APM.]

All subjects gave their informed written consent before participating in the study. Experiments were conducted in accordance with the Declaration of Helsinki and approved by the University of Salzburg’s institutional review board.

### Tasks

As second part of a larger study that otherwise included an attention task completely unrelated to the research question addressed in this manuscript, participants completed a subtraction task to assess number magnitude processing and a multiplication task to assess arithmetic fact retrieval during fMRI. 38 participants (20 men, 18 women) completed first the subtraction and then the multiplication task, 36 participants (20 men, 16 women) completed first the multiplication and then the subtraction task.

Stimuli were presented on an MR-compatible back-projection screen (Siemens CONCRAC 18.1”, resolution 1280 × 1024) using Presentation software (version 0.71, 2009, Neurobehavioral Systems Inc., Albany, CA, USA) on a Samsung HD322HJ PC. Both the subtraction and the multiplication task lasted for about 20 minutes. In both tasks, arithmetic problems were presented together with a solution probe in white on a black background (font size = 100, which corresponds to 2.75 cm on the screen). Participants were instructed to decide, whether the solution probe was correct or incorrect by pressing the left or right response button respectively on a fiber optic response device (Current Designs, HHSC-1×4-D). Responses were made with the right hand, i.e. participant’s dominant hand. In both, subtraction and multiplication, each item appeared with both correct and incorrect solution probes. Size and parity of solution probes was matched between subtraction (mean solution probe: 34.91 ± 5.07) and multiplication (mean solution probe: 35.00 ± 7.24), to ensure comparability of the two tasks. Behavioral data are openly available as Supplement 1.

#### Subtraction

Two-digit subtractions appeared in the center of the screen conjointly with a two-digit solution probe (e.g. 45–21 = 24). 64 different subtraction items were each presented twice – once with a correct and once with an incorrect solution probe. Incorrect solution probes differed from the correct responses by ±2 or ±10, such that participants could not base their decisions on the parity of the solution probe. In each item, subtrahend and minuend consisted of four different digits. Thus, participants completed a total of 128 subtraction items, which were presented in randomized order, intermitted by 32 null events (fixation cross). Each item was presented for 5 seconds, followed by a 2.5 seconds inter-stimulus interval, during which a fixation cross appeared on the screen.

Two factors that were previously identified as affecting subtraction performance were borrowing and distance e.g. ref 43. Decreased performance was observed in items requiring borrowing and items with a large distance between subtrahend and minuend. Therefore, across the 64 items, borrowing and distance were varied in a 2 × 2 design. Half of the items required borrowing (i.e. the unit digit of the minuend was larger than the unit digit of the subtrahend, e.g. 43–29), the other half did not require borrowing (i.e. the unit digit of the minuend was smaller than the unit digit of the subtrahend, e.g. 46–31). In large distance items, decade distance was larger than 4 (average distance 51.50 ± 10.08), in small distance items decade distance was smaller than 4 (average distance 18.33 ± 4.75). Size and parity of minuend, subtrahend, decade digit of minuend and subtrahend, as well as unit distance between minuend and subtrahend were all matched across the four categories (borrowing – large distance, borrowing – small distance, no borrowing – large distance, no borrowing – small distance).

#### Multiplication

Single-digit multiplications appeared in the center of the screen conjointly with a two-digit solution probe (e.g. 3 × 4 = 12). 64 different multiplication items were used and each was presented 4 times – twice with a correct solution probe and twice with an incorrect solution probe. Thus, participants completed a total of 256 multiplication items, which were presented in randomized order, intermitted by 64 null events (fixation cross) and 48 filler items, which were not included in the analysis. Filler items were presented to ensure that each related solution probe was presented equally often as correct or incorrect solution probe, such that participants could not base their decisions on the frequency of a number among correct or incorrect solution probes. Each item was presented for two seconds, followed by a 1 s inter-stimulus interval, during which a fixation cross appeared on the screen.

Two factors that were previously identified as affecting multiplication performance are relatedness and decade consistency of incorrect solution probes. It is harder to reject an incorrect solution probe, if it is related to the same multiplication series as the correct result or shares the same decade digit as the correct result^44^. Therefore, as in ref. 44, incorrect solution probes varied relatedness and decade-consistency in a 2 × 2 design. One of the incorrect solution probes was related to the same multiplication series as the correct result (e.g. 3 × 4 = 15), the other one was unrelated to the multiplication series of the correct result (e.g. 3 × 4 = 12). Furthermore, half of the related and unrelated solution probes were either decade consistent or inconsistent to the correct result. Decade consistent solution probes shared the same decade digit with the correct result (e.g. 3 × 4 = 15), whereas decade inconsistent solution probes contained a different decade digit than the correct result (e.g. 3 × 4 = 21). Size and parity of the first and second number, as well as size and parity of the solution probe and distance of the solution probe to the correct result were matched across the four categories (unrelated – consistent, unrelated – inconsistent, related – consistent, related – inconsistent). In each category, half of the incorrect solution probes were smaller than the correct result, the other half were larger than the correct result.

### fMRI data acquisition

Functional images as well as high resolution structural images were acquired on Siemens Magnetom TIM Trio 3 Tesla scanner (Siemens Healthcare). For functional images 36 transversal slices were taken oriented parallel to the AC-PC line using a T2*-weighted gradient echo planar (EPI) sequence (whole brain coverage, TE = 30 ms, TR = 2250 ms, flip angle 70°, slice thickness 3.0 mm, matrix 192 × 192, FOV 192 mm, in-plane resolution 2.6 × 2.6 mm). For structural images we used a T1-weighted 3D MPRAGE sequence (160 sagital slices, slice thickness = 1.2 mm, TE 2.9 ms, TR 2.3 ms, TI delay 900 ms, FA 0.95°, FOV 256 × 256 mm).

### fMRI data analysis

SPM8 (http://www.fil.ion.ucl.ac.uk/spm) standard procedures and templates were employed for analysis of functional images. The first six images of each session were discarded. Pre-processing steps were: (i) realignment and unwarping^45^, (ii) slice time correction, (iii) segmentation and normalisation of structural images to MNI standard stereotactic space (iv) co-registration of functional and structural images (v) normalisation of functional images using the parameters obtained in step (iii). To enhance activation detection, normalised functional images were resampled to isotropic 3 × 3 × 3 mm voxels and smoothed with a 6 mm Gaussian kernel.

A two stage mixed effects model was applied. At first level the parameter estimates for each subject and item category were calculated by a canonical hemodynamic response function in the context of a GLM. For each task we separately modelled responses to each category and null events. Only correctly solved trials were modelled. The six movement parameters were also included as regressors in the model. A high pass filter cut-off was set at 128 seconds and autocorrelation correction was performed using an AR(1) model^46^.

For both tasks contrast comparing all items to null events were defined at first level, as well as contrast modelling the borrowing effect (borrowing > no borrowing) and distance effect (large distance > small distance) in subtraction and the relatedness effect (related > unrelated) and consistency effect (consistent > inconsistent) in multiplication. Thus, for each task, three contrasts were defined at first level. Each contrast was compared between men and women at second level, using 2-samples t-tests. Furthermore, the task contrasts (Subtraction > null events; multiplication > null events) entered a flexible-factorial design with task as a within-subjects and sex as a group-factor in order to evaluate interactions between task and sex. Activation maps were thresholded at a voxel level threshold of p < 0.001 (uncorrected) and k > 80 voxels cluster size. The cluster-level FDR corrected p-value (threshold p < 0.05) was reported. fMRI data are openly available at http://webapps.ccns.sbg.ac.at/OpenData/.

### Hormone analysis

Participants gave two saliva samples, one before and one after entering the scanner. Saliva samples were stored at −20 °C until hormone assessment and centrifuged for 20 min at 3000 rpm. Estradiol, Progesterone and Testosterone were assessed using DeMediTec free in saliva ELISAs and were averaged over the two samples. Testosterone levels were significantly higher (t_(72)_ = 5.97, p < 0.001, d = 1.41) in men (121.65 ± 72.04 pg/ml) than in women (45.65 ± 18.59 pg/ml). Progesterone and estradiol levels were higher (E: t_(66)_ = 1.42, p = 0.16, d = 0.35, P: t_(72)_ = 5.05, p < 0.001, d = 1.19) in women (E: 2.59 ± 0.64 pg/ml, P: 185.68 ± 147.15 pg/ml) than in men (E: 2.32 ± 0.83 pg/ml, P: 59.07 ± 68.93 pg/ml). [Note that Estradiol values are not available for 6 participants due to insufficient sample volume].

## Results

### Behavioral results

#### Subtraction

**Figure 1:**
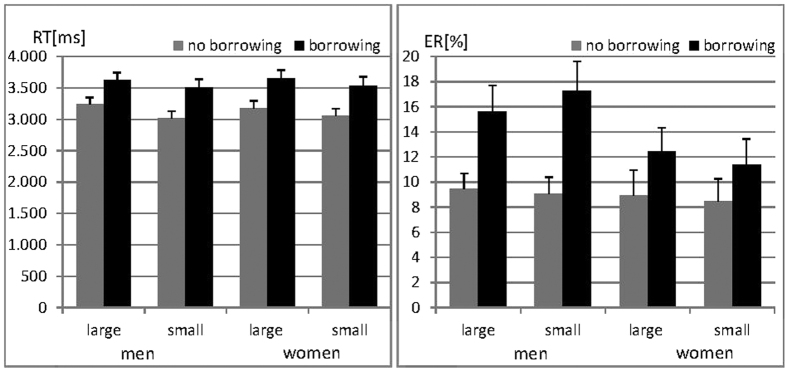
Reaction times (RT) and Error rates (ER) during subtraction in men and women. RT and ER were significantly higher for borrowing compared to non-borrowing items. RT, but not ER, were significantly higher for items with large compared to items with small distance between subtrahend and minuend. No significant sex differences were observed. The borrowing effect in ER was larger in men.

In order to confirm the borrowing and distance effects reported in the literature and evaluate sex differences on subtraction performance, 2 × 2 × 2 mixed ANOVAs with the within-subject factors *‘borrowing’* and *‘distance’* and the between subjects factor ‘*sex’* were performed on both reaction times (RT) and error rates (ER). Results are displayed in Fig. 1.

In both RT and ER a significant main effect of borrowing was confirmed (both F_(1,72)_ > 32.98, both p < 0.001, both η_p_^2^ > 0.31) with slower reactions and more errors for items requiring borrowing. A significant main effect of distance was only confirmed in the analysis of RT (F_(1,72)_ = 25.82, p < 0.001, η_p_^2^ = 0.26), but not ER (F_(1,72)_ = 0.01, p = 0.93, η_p_^2^ < 0.001). Reactions were faster for items with small distance than for items with large distance. There was no interaction between borrowing and distance in either the analysis of RT or ER (both F_(1,72)_ < 0.37, both p > 0.54, both η_p_^2^ < 0.005).

There was no main effect of sex on either RT or ER (both F_(1,72)_ < 1.24, both p > 0.24, both η_p_^2^ < 0.02). Sex did not interact with distance in the analysis of either RT or ER (both F_(1,72)_ < 0.95, both p > 0.33, both η_p_^2^ < 0.02) and also not with borrowing in the analysis of RT (F_(1,72)_ = 0.36, p = 0.55, η_p_^2^ = 0.005). There was however a significant interaction between sex and borrowing in the analysis of ER (F_(1,72)_ = 4.78, p = 0.03, η_p_^2^ = 0.06), such that the borrowing effect was larger in men than in women. Men and women did not differ in ER on items without borrowing, but men made more errors on items requiring borrowing.

#### Multiplication

**Figure 2:**
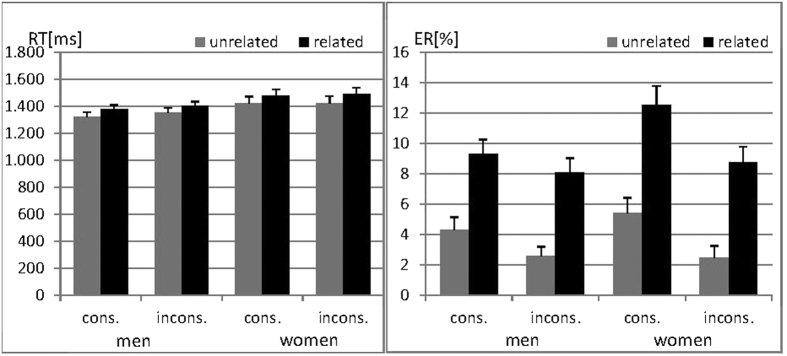
Reaction times (RT) and Error rates (ER) during multiplication in men and women. RT and ER were significantly higher for solutions related to the same multiplication series as the correct result compared to unrelated solution probes. ER, but not RT, were significantly higher for solution probes which shared the decade digit with the correct result (decade consistent, cons), than for decade inconsistent (incons) solution probes. No significant sex differences were observed.

In order to confirm the relatedness and decade consistency effects reported in the literature and evaluate sex differences on multiplication performance, 2 × 2 × 2 mixed ANOVAs with the within-subjects factors ‘*relatedness*’ and ‘*decade consistency*’ and the between subjects factor ‘*sex*’ were performed on both RT and ER. Results are displayed in Fig. 2.

In both RT and ER a significant main effect of relatedness was confirmed (both F_(1,72)_ > 119.95, both p < 0.001, both η_p_^2^ > 0.62) with slower reactions and more errors in items with related solution probes, i.e. solution probes from the same multiplication series as the correct result. A significant main effect of consistency was confirmed only for ER (F_(1,72)_ = 21.75, p < 0.001, η_p_^2^ = 0.23), but the effect was non-significant for RT (F_(1,72)_ = 3.47, p = 0.07, η_p_^2^ = 0.05). Participants made significantly more errors with consistent solution probes, i.e. solution probes containing the same decade digit as the correct result. Relatedness and consistency did not interact in either the analysis of RT or ER (both F_(1,72)_ < 0.09, both p > 0.77, both η_p_^2^ < 0.001).

There was no main effect of sex on either RT or ER (both F_(1,72)_ < 2.45, both p > 0.12, both η^2^ < 0.04). In the analysis of RT, sex did not interact with either the relatedness or consistency effects (both F_(1,72)_ < 1.19, both p > 0.27, both η_p_^2^ < 0.02). Both interactions were also non-significant in the analysis of ER (both F_(1,72)_ < 3.35, both p > 0.07, both η_p_^2^ < 0.05).

#### Comparison of subtraction and multiplication

In order to compare multiplication and subtraction 2×2 mixed ANOVAS with the within-subjects factor task and the between-subjects factor sex were performed on both RT and ER.

For reaction times a significant main effect of task (F_(1,72)_ = 822.67, p < 0.001, η_p_^2^ = 0.92), but no effect of sex and no task × sex interaction were observed (both F_(1,72)_ < 0.42, both p > 0.51, both η_p_^2^ < 0.006), indicating that reactions were significantly faster in multiplication than in subtraction for both men and women.

For error rates, a significant main effect of task was observed (F_(1,72)_ = 22.86, p < 0.001, η_p_^2^ = 0.24), indicating that participants made more errors in subtraction than in multiplication. The main effect of sex was non-significant (F_(1,72)_ = 0.20, p = 0.66, η_p_^2^ = 0.003) and there was no significant task × sex interaction (F_(1,72)_ = 3.47, p > 0.06, η_p_^2^ = 0.05).

Reaction times, as well as error rates were significantly correlated between subtraction and multiplication in both men and women (all r > 0.43, all p < 0.01), indicating that participants with better performance in one task also displayed better performance in the other task. In RT, correlation between subtraction and multiplication was significantly higher in women (r = 0.75) than in men (r = 0.52), as indicated by Fisher’s z transformation (z = 1.68, p < 0.05).

### Neuroimaging results

#### Subtraction

**Figure 3:**
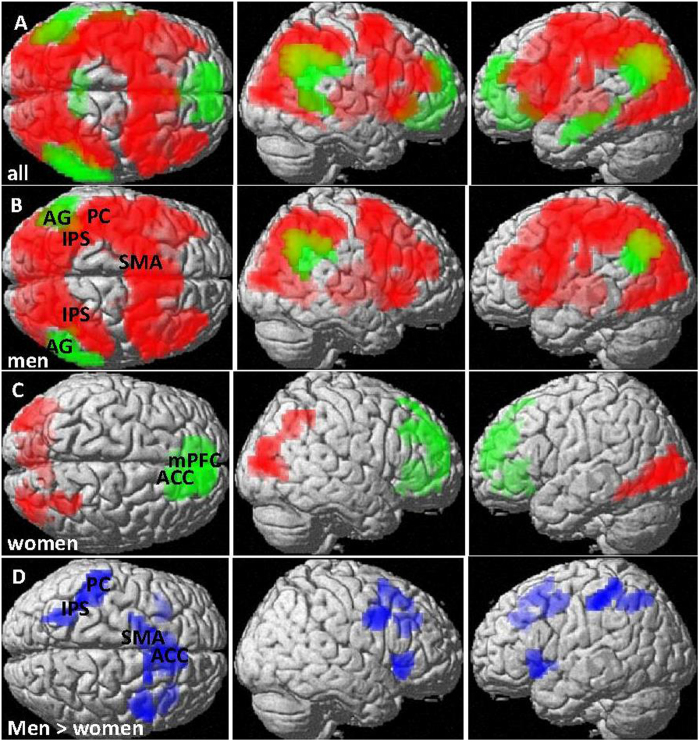
BOLD-response to subtraction (number magnitude processing). (**A**) all participants, (**B**) men and (**C**) women. Red: Activation areas, Green: deactivation areas. (**D**) Areas with significantly higher BOLD-response (more activation/less deactivation) in men compared to women (blue). Activation in central and parietal areas including the IPS was significantly higher in men compared to women. Pictures are thresholded at p_FDR_ < 0.05 (cluster level). Primary threshold was p < 0.001 for (**A**,**B**,**D**), but p < 0.005 for (**C**). AG = angular gyrus, IPS = intra-parietal sulcus, mPFC = medial prefrontal cortex, ACC = anterior cingulate cortex, PC = postcentral gyrus, SMA = supplementary motor area.

Over all participants, subtraction activated a large centro-parieto-occipital network (Fig. 3A) with a global peak in the left IPS ([−45, −40, 58], T = 6.79, k = 12645 voxels, p_FDR_ < 0.001) and yielded deactivations in the bilateral AG/SMG (left: [−60, −58, 25]; T = 5.38, k = 400 voxels, p_FDR_ < 0.001; right: [54, −61, 43], T = 5.70, k = 635 voxels, p_FDR_ < 0.001), and the medial prefrontal cortex (mPFC)/anterior cingulate gyrus (ACC) ([0, 23, −11], T = 5.02, k = 1002 voxels, p_FDR_ < 0.001). This pattern was largely attributable to men (Fig. 3B), since clusters of significant BOLD-response to subtraction in women could only be confirmed at a more liberal primary threshold of p = 0.005 (Fig. 3C). Specifically among deactivation sides, the AG/SMG could only be confirmed in men, whereas the mPFC/ACC could only be confirmed in women.

In direct comparison (Fig. 3D), men displayed significantly stronger activation than women in the left IPS and postcentral gyrus ([−42, −43, 61], T = 4.12, k = 223 voxels, p_FDR_ = 0.007), the left and right Insula (left: [−36, 20, −8], T = 4.66, k = 103 voxels; p_FDR_ = 0.056; right: [42, 23, −2], T = 4.36, k = 100 voxels, p_FDR_ = 0.056), the right precentral gyrus ([42, 5, 34], T = 4.89, k = 129 voxels, p_FDR_ = 0.04), the left supplementary motor area ([−12, 17, 49], T = 4.58, k = 232 voxels, p_FDR_ = 0.007) and less deactivation than women in the ACC ([9, 38, 34], T = 4.16, k = 145 voxels, p_FDR_ = 0.03).

No borrowing or distance effects were observed in either activation or deactivation and no sex differences in the borrowing or distance effects in BOLD-response were observed.

#### Multiplication

**Figure 4:**
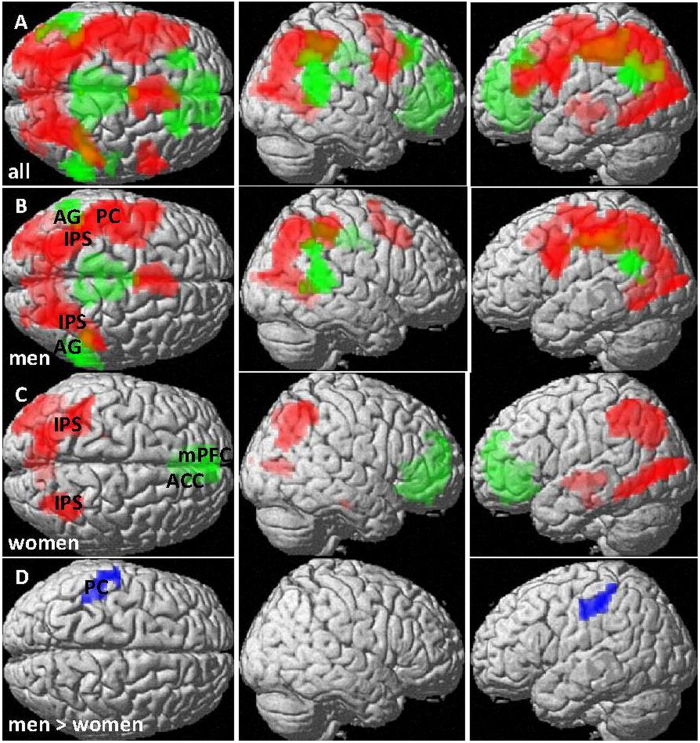
BOLD-response to multiplication (arithmetic fact retrieval). (**A**) all participants, (**B**) men and (**C**) women. Red: Activation areas, Green: deactivation areas. (**D**) Areas with significantly higher BOLD-response (more activation/less deactivation) in men compared to women (blue). Activation in the left AG/SMG was significantly higher in men compared to women. Pictures are thresholded at p_FDR_ < 0.05 (cluster level). Primary threshold was p < 0.001 for (**A**,**B**,**D**), but p < 0.005 for (**C**). AG = angular gyrus, IPS = intra-parietal sulcus, mPFC = medial prefrontal cortex, ACC = anterior cingulate cortex, PC = postcentral gyrus.

Over all participants, multiplication activated a large left-lateralized centro-parieto-occipital network (Fig. 4A). Deactivations were observed in the bilateral AG/SMG (left: [−57, −55, 25], T = 4.92, k = 229 voxels, pFDR = 0.002), the mPFC/ACC ([−12, 50, 1], T = 5.43, k = 914 voxels, pFDR < 0.001) and the Precuneus ([24, −40, 22], T = 4.61, k = 566 voxels, pFDR < 0.001). Again, this pattern was largely attributable to men (Fig. 4B), while activation was less extensive in women (Fig. 4C). Again, the AG/SMG could only be confirmed as deactivation site in men, whereas the mPFC/ACC could only be confirmed in women. In direct comparison (Fig. 4D), men displayed significantly stronger activation in the left postcentral gyrus ([−60, −22, 43], T = 4.58, k = 84 voxels, p_FDR_ = 0.02) than women.

No relatedness or consistency effects were observed in either activation or deactivation areas and no sex differences in the relatedness or consistency effects in BOLD-response were observed.

#### Comparison of subtraction and multiplication

**Figure 5:**
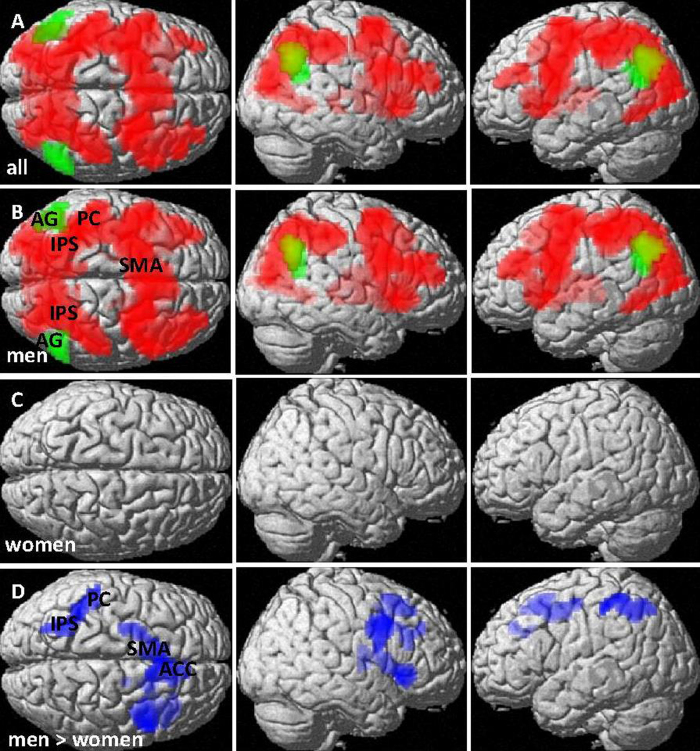
Comparison of BOLD-response to subtraction (number magnitude processing) and multiplication (arithmetic fact retrieval). (**A**) all participants, (**B**) men and (**C**) women. Red: Subtraction > multiplication, Green: multiplication > subtraction. (**D**) Areas with significantly stronger subtraction > multiplication contrast in men compared to women (blue). In men, subtraction yielded stronger activation than multiplication in a large network including the IPS, while multiplication yielded stronger activation than subtraction in the AG/SMG. In women, no difference was observed in BOLD-response between subtraction and multiplication. Pictures are thresholded at p_FDR_ < 0.05 (cluster level). Primary threshold was p < 0.001 for (**A**,**B**,**D**), but p < 0.005 for (**C**). AG = angular gyrus, IPS = intra-parietal sulcus, mPFC = medial prefrontal cortex, ACC = anterior cingulate cortex, PC = postcentral gyrus, SMA = supplementary motor area.

Over all participants, subtraction yielded significantly stronger activation than multiplication in a large centro-parieto-occipital network including the bilateral IPS, whereas multiplication yielded significantly less deactivation than subtraction in the AG bilaterally (left: [−51, −70, 34], T = 4.85, 190 voxels, p_FDR_ = 0.006; right: [54, −64, 37], T = 4.76, k = 155 voxels, p_FDR_ = 0.008; Fig. 5A). This pattern of results was largely attributable to the male sample (Fig. 5B) since no sites of differential activation between subtraction and multiplication could be confirmed for the female sample (Fig. 5C), not even at a highly liberal primary threshold of p < 0.01 (uncorrected).

Sex differences in the Subtraction vs. Multiplication contrast reached significance in the mPFC/ACC ([9, 38, 34], T = 4.65, k = 122 voxels, p_FDR_ = 0.02), the supplementary motor area ([−12, 17, 49], T = 4.87, k = 214 voxels, p_FDR_ = 0.005), the right precentral gyrus ([42, 8, 34], T = 5.14, k = 200 voxels, p_FDR_ = 0.005), the right Insula ([42, 23, −2], T = 4.32, k = 197 voxels, p_FDR_ = 0.003), as well as the left IPS ([−27, −61, 49], T = 3.98, k = 172 voxels, p_FDR_ = 0.007) with stronger differences in men compared to women (Fig. 5D).

## Discussion

The present study was designed to identify, whether the dissociability of neural systems for number magnitude processing and arithmetic fact retrieval can be confirmed for both, men and women. We utilized a subtraction task to assess number magnitude processing and a multiplication task to assess arithmetic fact retrieval. In both tasks, previously demonstrated behavioral effects could be replicated for both, men and women with some indication that the size of the effects might be modulated by sex.

In men, we found the expected segregation of neuronal systems with stronger activation for subtraction than multiplication in a large network including the intraparietal sulcus (IPS) and stronger activation for multiplication than subtraction in the AG/SMG, although bilaterally. This replicates findings of Lee *et al*.^2^, even though they used single-digit subtraction instead of two-digit subtraction^2^. However, no sites of differential activation in subtraction and multiplication were observed in women.

These sex differences in the overlap between subtraction and multiplication were largely attributable to sex differences in the activation patterns during subtraction. While men activated a large centro-parieto-occipital network while solving subtraction items, only weak activation was observed in women in the occipital cortex (compare Fig. 3C). These were essentially the same activation patterns as during multiplication. Furthermore, while during both tasks deactivation in men was centered in posterior parts of the inferior parietal lobules (AG/SMG), deactivation in women was centered in the medial prefrontal cortex/anterior cingulate gyrus (mPFC/ACC). Differences in deactivation sites during number magnitude processing between men and women have previously been observed^47^.

The results of the present study compare nicely to the results of our previous study on sex differences during number bisection^38^. As during number bisection, brain activation patterns differed between number magnitude processing and arithmetic fact retrieval only in men. As during number bisection, sites of differential deactivation between men and women were the AG/SMG and mPFC/ACC. Furthermore, as during number bisection, only men showed performance differences between number magnitude processing (non-multiplicative bisection items, subtraction) and arithmetic fact retrieval (multiplicative bisection items, multiplication). Stronger activation of the IPS and AG in men compared to women during a combination task of addition (fact retrieval) and subtraction (magnitude processing) was also observed in a neuroimaging study of Keller & Menon (2009), although in their study the differences were centered in the right hemisphere^48^.

Like in the present study none of the previous neuroimaging studies on number processing observed overall behavioral differences in task performance between men and women^38^,^39^,^48^, even though sex differences in performance have been suggested in a variety of behavioral studies e.g.^36^,^37^. While sampling differences may account for this variation it is remarkable to note that comparable sex differences in the neural correlates of number processing were observed in all tasks. This suggests that women recruit the neural substrates of number processing differently than men in order to reach a similar level of performance.

It has previously been demonstrated that women avoid the use of spatial strategies and utilize verbal and memory strategies to solve spatial tasks and reach comparable performance to men^49^,^50^. This is reflected in differential brain activation during spatial processing between men and women with unique frontal activation in women^51^,^52^,^53^,^54^. Particularly in stressful testing situations, such as in a scanning environment, women may be inclined to choose a less effortful processing strategy and avoid spatial processing. This could also explain the lack of behavioral differences in neuroimaging studies of number processing, but also of spatial processing. The lack of parietal activations during subtraction in women, suggests a similar avoidance of spatial processing during subtraction in the present study. This could account for the similarity between brain activation during subtraction and multiplication in women.

It is also possible that there is a larger range of different strategies used to solve the two tasks in the female sample. Assuming that a larger range of strategies results in stronger variation in brain activation patterns, this could account for the weak activation patterns to subtraction and multiplication observed in women. It is however also possible that this lack of distinct activation patterns is due to a lack of statistical power in the female sample.

In summary the neural substrates for subtraction and multiplication differ as expected in the male sample, but largely overlap in the female sample. Moreover, the results in the whole sample were largely driven by the strong differences in the male subsample, which explains why similar results have previously been obtained in mixed sex samples. It can be concluded that while behavioral evidence on sex differences in numerical abilities is still inconsistent, the neuronal systems supporting number processing differ between men and women, as may the strategies men and women employ to solve different numerical tasks. Thus, sex and hormonal status should be taken into account when studying the neural correlates of number processing.

## Additional Information

**How to cite this article:** Pletzer, B. Sex differences in number processing: Differential systems for subtraction and multiplication were confirmed in men, but not in women. *Sci. Rep.* **6**, 39064; doi: 10.1038/srep39064 (2016).

**Publisher's note:** Springer Nature remains neutral with regard to jurisdictional claims in published maps and institutional affiliations.

## Supplementary Material

Supplementary Dataset 1
